# 

**Published:** 1820-12-01

**Authors:** 


### *Books received for Review since last Quarter.*

1. *An Historical Account of St. Thomas's Hospital, Southwark.* By Benjamin Golding, Member of the Royal College of Surgeons, London, &c. &c. One vol. small octavo, pp. 245. London, 1820.

☞ *This account, drawn up with great learning, research, and apparent fidelity, cannot fail to prove highly interesting to all those who have received their education at, or are otherwise connected with, the venerable and useful Institution abovementioned.*

---

2. *Report on the Epidemic Cholera Morbus, as it visited the territories subject to the presidency of Bengal, in the years* 1817, 1818, *and* 1819. *Drawn up by order of the Government, under the superintendence of the Medical Board.* By James Jameson, assistant surgeon and secretary to the Board. One vol. octavo, pp. 325. Calcutta, 1820.

☞ *For this interesting report we are indebted to Mr. William Evans, of the Royal Navy, lately returned from the East. Some account of it will be seen in the present No.*

3. *Remarks on the Importance of the Medical Profession, and on the present State of Medical Practice in Ireland.* By RICHARD GRATTAN, M. D. Fellow and Censor of the College of Physicians, Ireland. Octavo. Dublin, 1820. Parts I. and II. P. 70 and 50.

---

4. *Elements of the Theory and Practice of Physic, designed for the use of Students. Part* I. *including the Symptoms, Pathology, and Treatment of Acute Diseases.* By GEORGE GREGORY, M. D. Licentiate of the Royal College of Physicians, London, and Senior Physician to St. George's and St. James's Dispensary. One vol. octavo, pp. 401. London, 1820.

☞ *See our present No. p.* 435.

---

5. *Lectures on the Structure and Physiology of the Parts composing the Skeleton, and on the Diseases of the Bones and Joints of the Human Body, preceded by some Observations on the Influence of the Brain and Nerves, delivered before the Royal College of Surgeons of London, in the Summer of* 1820. By JAMES WILSON, F. R. S. &c. &c. &c. One vol. octavo, pp. 414. London, 1820.

☞ *See our present No. p.* 427.

---

6. The Principles of Midwifery; including the Diseases of Women and Children. By JOHN BURNS, C. M. Regius Professor of Surgery in the University of Glasgow, &c. *Fifth* edition, enlarged, pp. 755. London, 1820.

---

7. A Synopsis of the various kinds of difficult Parturition, with practical Remarks on the Management of Labours. *Third edition,* with considerable additions, and an Appendix of illustrative Cases and Tables. By S. MERRIMAN, M. D. F. L. S. Lecturer on Midwifery, &c. &c.

☞ *See page* 526 *of this Number.*

---

8. On the Elasticity of the Lungs. By JAMES CARSON, M. D. Quarto, pp. 16, with a plate. (From the Phylosophical Transactions.) London, 1820.

---

9. A Treatise on the Plague, designed to prove it contagious, from facts, collected during the Author's residence in Malta, when visited by that Malady in 1813. With Observations on its Prevention, Character, and Treatment; to which is annexed an Appendix, containing minutes of the Author's evidence, given before the Contagion Committee of the House of Commons, accompanied by their Report. By SIR ARTHUR BROOKE FAULKENER, M. D. Fellow of the Royal College of Physicians, &c. &c. One vol. octavo, with plans, pp. 412. London, 1820.

10. An Address to Persons afflicted with Deafness, particularly the obscure cases, denominated Nervous Deafness, with Comments, &c. By W. Wright, Surgeon-Aurist in extraordinary to Her late Majesty. One vol. small octavo, pp. 97. London, 1820.

---

11. Memoires sur la fièvre jaune, considerée dans sa Nature et dans ses rapports avec les Gouvernment. Par N. V. A. Gerardin, de Naucy, Docteur en Medecine, &c. Octavo, pp. 91. 1820. *Transmitted by M. Breschet.*

---

12. De la Goutte et des Maladies Goutteuses; to which is added, Recherches Pratiques sur le Rheumatisme, traduit de l'Anglais de James Johnson, M. D. Auteur de l'ouvrage intitulé—"*Influence of Tropical Climates on European Constitutions;*" et Redacteur du "*Medico-Chirurgical Journal.*" Par J. N. Guilbert, M. D. One vol. octavo. Paris, 1820.

---

13. An Essay on Mineral, Animal, and Vegetable Poisons, in which the symptoms, mode of treatment, and tests of each particular poison, with the general morbid appearances on dissection, are concisely detailed. To which is added, an Account of the Means to be employed in cases of suspended Animation. Small duodecimo, pp. 67, with ten plates of the poisonous plants. 1820.

☞ *This appears to be a convenient pocket companion, as the toxicological chart is a tenant of the shop or library.*

---

14. A Chemical and Medical Report of the Properties of the Mineral Waters of Buxton, Matlock, Tunbridge-Wells, Harrogate, Bath, Cheltenham, Leamington, Malvern, and the Isle of Wight. By Charles Scudamore, M.D. Member of the Royal College of Physicians, &c. One vol. octavo, pp. 265. London, 1820.

---

15. A few Hints relative to Cutaneous Complaints. By T. M. Kelson. A new edition, with some Remarks on the Nature and Functions of the Skin. By C. Kelson, Member of the Royal College of Surgeons, &c. Octavo, sewed, pp. 31, 1820.

☞ *These "few Hints" relate to a secret method of curing Diseases of the skin. We have nothing to do with such works.*

---

16. American Medical Recorder, for January, April, and July, 1820; conducted by John Eberle, M. D. &c. assisted by several eminent practitioners; in exchange for the Medico-Chirurgical Review.

---

17. New York Medical Repository, up to June 1820, in exchange for Med. Chir. Review.

18. Revue Medicale, Historique et Philosophique, No. IV. for July, 1820, and No. V. for September, in exchange for MED.-CHIR. REVIEW.

---

19. Journal General de Medicine, &c. for 1820, up to September. In exchange for the MED. CHIR. REVIEW.

---

20. Pharmacologia; or, the History of Medicinal Substances, with a view to establish the art of prescribing and of composing extemporaneous formulæ, upon fixed and scientific principles; illustrated by formulæ, in which the intention of each element is designated by key letters. By JOHN AYRTON, Paris, M.D. F.L.S. M.R.I. Fellow of the Royal College of Physicians of London, &c. &c. &c. One vol. octavo, pp. 580, *fourth edition*, much enlarged. London, 1820.

---

21. Views of the Muscles of the Human Body, drawn from Nature, and engraved by George Lewis; accompanied by suitable explanatory references. Designed as a guide to the student of anatomy, and a book of reference for the medical practitioner. *One volume, quarto, containing eighteen plates, price 1l. 11s. 6d. boards. Highly,* 1820.

*The Author dissected as an Artist, and his delineations appear to us to be very correct and expressive. Among the subscribers to this Work, we see the very respectable names of Abernethy, Brodie, Brookes, Cline, Carlisle, Astley Cooper, Earle, Grainger, Guthrie, Green, Latham, Lawrence, Monro, Mayo, Powel, Stanley, Taunton, Travers, Uwins, Wilson, &c.* We need not say that these names confer respectability on any work to which they are prefixed.

---

22. Considerations et Observations Anatomiques et Chirurgicales sur la Formation, la Disposition, et le Traitment des Fistules Stercorales, et des Anus-Contre-Nature. Par GILBERT BRESCHET, Docteur en Medecine, Chef des Travaux Anatomiques de la Faculté de Medecine de Paris; premier Aide de Clinique a l'Hotel-Dieu, &c. &c. Paris, 1820. *In manuscript.* Premier partie, 77 pages.

☞ This important manuscript has been presented to us by our able and esteemed friend, M. Breschet, anterior to its appearance in France. The plan of our Journal not admitting original communications, we have presented it to our able and respected cotemporary, "*the Quarterly Journal of Foreign Medicine and Surgery*," after its publication in which we shall prepare an analysis of it for our Supplemental Review.

---

23. Outlines of Midwifery, developing its principles and practice; with twelve lithographic engravings. By J. T. CONQUEST, M.D. F.L.S. Member of the Royal College of Physicians; Physician-Accoucheur to the City of London Lying-in-Institution, &c. &c. One vol. duodecimo, pp. 193. London, 1820.

24. A practical Treatise on the Diseases of the Eye. By John Vetch, M. D. F. R. S. E. Member of the Royal Medical Society of Edinburgh, and the Medico-Chirurgical Society; lately Physician to the Forces, and principal Medical Officer to the Ophthalmia Military Hospital. Octavo, pp. 266, and three coloured plates, London, 1820.

---

25. General Elements of Pathology. By Whitlock Nichol, M. D. M. R. I. A. F. L. S. of the Royal College of Physicians, London, &c. One vol. octavo, pp. 234. London, 1820.

---

26. Synopsis Nosologica Morborum, quibus Infantes ac Pueri tentantur, Legibus Physiologicis recensita; et in Noscomii Regalis ad Morbos presertim Puerorum debellandos. Ab Augusto B. Granville, M. D. S. R. S. Statuta. in the form of a Chart. 1820.

---

27. The New-England Journal of Medicine and Surgery, and Collateral Branches of Science. Conducted by a number of Physicians. New Series, No. IV. Vol. IV. for October 1820. In exchange of Med. Chir. Review.

---

28. A Sketch of the Physiology and Pathology of Urine; with an historical Introduction. By Jonathan Osborne, M. B. T. C. D. Licentiate of the King's and Queen's College of Physicians in Ireland. Octavo, pp. 81. London, Nov. 1820.

---

29. Practical Observations on the Colchicum Autumnale, as a general remedy of great power, in the treatment of Inflammatory Diseases, both Acute and Chronic; and therefore as a substitute for bleeding in disorders which are connected with increased action of the heart and arteries. By Charles Thomas Haden, Esq. Surgeon to the Chelsea and Brompton Dispensary; late Surgeon to the Derbyshire General Hospital, &c. One vol. octavo, pp. 84. London, November, 1820.

---

30. Copy of the Report to the Secretary of State for the Home Department, dated 20th May, 1820.

*This Report came too late, and is too long, for insertion in a* Review; *we shall, however, present an abstract of it in our next number.* Edit.

---

31. An Historical and Practical Treatise on the Internal Use of the Hydro-cyanic (Prussic) Acid, in Pulmonary Consumption and other Diseases of the Chest; as well as in several Complaints attended by great Nervous Irritation or Acute Pain; with full directions for the preparation and administration of that medicine; and a preliminary descriptive account of the principal diseases in which it has been employed—illustrated by numerous cases. Second Edition, greatly enlarged. By A. B. Granville, M.D. F.R.S. F.L.S. M.R.I. Physician in Ordinary to his Royal Highness the Duke of

Clarence; Member of the Royal College of Physicians; Principal Physician to the Royal Infirmary for the Diseases of Children; and Physician-Accoucheur to the Westminster General Dispensary. One vol. p. 417. London, November, 1820.

---

To the authors of the foregoing works we return our best thanks, and beg to say, that such as are not reported on in the present No. are placed in proper hands for speedy analysis. No work of importance that appears in this country shall fail to be carefully analyzed in this Journal with all possible dispatch.

And here we are induced to make a remark or two in answer to an objection that has been thrown out against extensive analyses of medical works. The above list contains the titles of those works *only* which have been transmitted to us for review since last quarter. Many others have been published within the period stated. Now we ask this question; is there more than one in twenty of the readers of this journal, whose opportunities, leisure, or finances can extend to the purchase and perusal of the various works that issue from the medical press? We believe there is not one in fifty. And if this be the case, can the utility or importance of an Analytical Review, on a scale commensurate with the state of medical literature, be called for a moment in question? But we will go farther, and ask yet another question. Does not that twentieth or fiftieth person, who carefully peruses *all* medical works, find it very convenient to have also a concentrated analytical record of them, in a shape and size that may offer facility of re-perusal, and materials for reminiscence? We leave the answer to our readers.

---

We have been requested by Dr. Mott to give publicity to the following passage:—

*Further Remarks on the Case of Ligature of the Arteria Innominata.* By VALENTINE MOTT, M. D.

In my first account of this case, it is stated that, "*the subclavian artery, internally and externally to the disease, was pervious.*" To this it may now be added, that where the artery opens into the ulcer left from the wound of the operation, it appears not only pervious, but of the natural size, and the coats free from any diseased appearance. Externally towards the axilla the artery is somewhat enlarged in diameter, but exhibits no appearance of disorganization of its coats either externally or internally. About an inch from the ulcer, or just as the artery has passed between the scaleni muscles, there is an irregularly shaped eliptical opening upon its upper side, large enough to receive the extremity of the fore-finger. The edges of this opening are jagged and uneven, and the surface of the artery internally is of a brownish yellow colour, to the extent of half an inch on the inside of the opening, and more than an inch towards the axilla. The internal coat of the artery has a rugous or puckered appearance, separated a little from the muscular coat, very friable, and evidently in a degenerated state. This opening of the artery communicates directly with the anterior extremity of the sac, which contains coagula; and upon removing these, the surface of the sac is seen puckered or thrown into a great number of little folds, giving it at first sight the appearance of containing a number of holes. This account is taken from the morbid parts before me, and the preparation has been seen and examined by Dr. Post, Dr. Hosack, Dr. Watts, Dr. Stevens, and others, who have authorised me to state that they are satisfied of its aneurismal character.

---

We are happy to learn that Drs. Paris and Harrison are now delivering lectures on the materia medica, therapeutics, and pathology, which are likely to prove very advantageous to medical students in the West end of the town. We have had an opportunity of observing the mode in which the subjects are discussed, and we are of opinion, that the plans adopted by these gentlemen conduce much to the facility of acquiring pharmacological, therapeutical, and pathological knowledge.

---

In the press, a new (being the third) edition of Dr. Johnson's work on Tropical Climates. The diseases of the *Western* hemisphere will be investigated with great minuteness, and all the light which the last twenty years have elicited respecting them, will be collected into a focus in this edition. A physician of great talents, and long resident in the West Indies, is assisting the author in arranging and enriching this enlarged division of the Work. Great and important additions will be made to the *Eastern* division, and the whole Work is undergoing a thorough revision. It will be published in February, 1821.

---

### *Additional Subscribers since September.*

Abell, Dr. Brighton
Addison, Dr. Hatton Garden
Allinson, Mr. J. H. Surgeon, Woolwich
Ashford, Medical Society, Kent
Bailey, Mr. Surgeon, Blackburne
Bailey, Mr. Sam. Surgeon, Cape of Good Hope
Belcombe, Dr. York.
Blood, Mr. Westminster Hosp.
Butini, Dr. Adolphe, Geneva
Carrol, William, Esq. Surgeon, Walworth Road.
Clifford, Surgeon, Trim, Ireland
Cock, Mr. Easingwould [stated)
Cowen, Dr. Ireland, (town not
Curtis, Mr. Lisson-st. New-road
Dempster, Dr. (place not mentioned)
Hagley, Mr. Apothecary to the Oxford Infirmary.
Hall, Mr. Grosvenor-st. Westm.
Henderson, Mr. Surgeon, Barton, Bristol.
Holst, Dr. Public Health-officer, Christiana, Norway
Hodson, Mr. Surgeon, Lewes
Horsley, Dr. William, North Shields
Hurst, Mr. James C. Surgeon, Dartford, Kent
Hunter, Mr. Bishopsgate-street
Jesson, Dr. Germany
Jones, J. J. Esq. Margaret-street
Ives, Mr. Chertsey
Kingsley, Surg. Roscrea, Ireland
King, Mr. G. Surgeon, H. C. Service, Bengal

Drake, Richard, Esq. York
Duncan, Mr. Torrington-place
Editor of New Monthly Magazine, in exchange
Elmslie, Mr. Berners-street
Evans, Mr. William, Surg. R.N.
Flanagan, J. B. Esq. Surgeon of 9th Reg. Vet. Batt.
Foulkes, Edward, Esq. Surgeon, Eleven-towns
Geddes, Mr. A. Noble, Surgeon
Gibbs, Mr. Surgeon, Westbury, Wilts
Gissing, George Edward, Esq. Bury St. Edmunds
Greenish, Mr. John, Surgeon, R. N. Sheerness
Mitchel, J. Esq. Assistant Surgeon, 48th Regiment
Morrison, W. Esq. Surgeon, 90th Light Infantry, Malta
Napper, Mr. James, Surgeon R. N. Paris
Nason, Dr. Ireland, (place not stated)
Pascalis, Felix, M.D. New York, in exchange for New York Medical Repository
Peebles, Surgeon, Ireland, (place not stated
Plumb, Mr. Great Russel-street
Proud, John Freer, Esq. Member of the Royal College of Surgeons, and Surgeon to His Royal Highness the Duke of Gloucester, Wolverhampton
Pyper, Dr. 4th Dragoon Guards
Scaife, Mr. Coxwould
Smith, Dr. J. Gordon, Cleveland House, St. James's
Simson, Mr. Knaresbro'
Lake, Mr. A. P. Surg. R. N. Teignmouth, Devon.
Lambert, Mr. Surgeon, Thursk.
M'Donnel, J. (M.D.) 55 Regt. Infantry.
M'Kenzie, Dr. Assist. Surgeon to the Fever Hospital, Great Ormond-street
M'Rae, Mr. John, R. N. Surg. Old Broad Street
Malcom, Dr. J. Perth.
Martin, Dr.
Mauger, Mr. John, Surgeon, Guernsey
Miller, Mr. James, Perth
Milne, Garden, M. D. Bamffe
Moran, Francis, M. D. Ireland
Southey, Dr. Physician to the Middlesex Hospital
Strang, Mr. John, Surgeon Bridport
Tarn, Mr. John, Surgeon, H.M.S. Satellite, East Indies
Todd, Dr. 83d Regiment
Vaughan, Dr. Rochester
Wells, Silas, Esq. Bourton on the Water, Gloucestershire
White, W. R. Esq. 84th Regt.
Wilson, W. Esq. Aylbroth, Scotland
Williamson, James, Esq.
Winborne, Medical Society, Dorsetshire
Woodman, Mr. R. Surgeon, Weymouth
Wood, Sir James Athol, Albany, Piccadilly
Woodcock, Mr. John, Surgeon, Bury, Lancashire
Woodyer, C. Esq. Guilford

---

*Change of Residence, Appointments, &c. &c. &c.*

Sir Henry Halford, Bt. elected President of the Royal College of Physicians.
Dr. Harrison, from Sackville-street, to Argyl-street
Dr. Archibald Robertson, elected to the Northampton General Hospital.

---

☞ *A few Subscribers' names arrived too late. The list is put to press on the 20th of the month preceding publication.*

No. 4 will be published on the 1st *March*, 1821.

